# Monitoring Immune Checkpoint Regulators as Predictive Biomarkers in Hepatocellular Carcinoma

**DOI:** 10.3389/fonc.2018.00269

**Published:** 2018-07-13

**Authors:** Ritu Shrestha, Prashanth Prithviraj, Matthew Anaka, Kim R. Bridle, Darrell H. G. Crawford, Bijay Dhungel, Jason C. Steel, Aparna Jayachandran

**Affiliations:** ^1^Faculty of Medicine, The University of Queensland, Brisbane, QLD, Australia; ^2^Gallipoli Medical Research Institute, Greenslopes Private Hospital, Brisbane, QLD, Australia; ^3^Fiona Elsey Cancer Research Institute, Ballarat, VIC, Australia; ^4^Department of Medicine, University of Alberta, Edmonton, AB, Canada

**Keywords:** hepatocellular carcinoma, epithelial-to-mesenchymal transition, immune checkpoints, programmed death receptor ligand-1, immune modulation

## Abstract

The global burden of hepatocellular carcinoma (HCC), one of the frequent causes of cancer-related deaths worldwide, is rapidly increasing partly due to the limited treatment options available for this disease and recurrence due to therapy resistance. Immune checkpoint inhibitors that are proved to be beneficial in the treatment of advanced melanoma and other cancer types are currently in clinical trials in HCC. These ongoing trials are testing the efficacy and safety of a few select checkpoints in HCC. Similar to observations in other cancers, these immune checkpoint blockade treatments as monotherapy may benefit only a fraction of HCC patients. Studies that assess the prevalence and distribution of other immune checkpoints/modulatory molecules in HCC have been limited. Moreover, robust predictors to identify which HCC patients will respond to immunotherapy are currently lacking. The objective of this study is to perform a comprehensive evaluation on different immune modulators as predictive biomarkers to monitor HCC patients at high risk for poor prognosis. We screened publically available HCC patient databases for the expression of previously well described immune checkpoint regulators and evaluated the usefulness of these immune modulators to predict high risk, patient overall survival and recurrence. We also identified the immune modulators that synergized with known immune evasion molecules programmed death receptor ligand-1 (PD-L1), programmed cell death protein-1 (PD-1), and cytotoxic T lymphocyte-associated antigen-4 (CTLA-4) and correlated with worse patient outcomes. We evaluated the association between the expression of epithelial-to-mesenchymal transition (EMT) markers and PD-L1 in HCC patient tumors. We also examined the relationship of tumor mutational burden with HCC patient survival. Notably, expression of immune modulators *B7-H4, PD-L2, TIM-3*, and *VISTA* were independently associated with worse prognosis, while *B7-H4, CD73*, and *VISTA* predicted low recurrence-free survival. Moreover, the prognosis of patients expressing high *PD-L1* with high *B7-H4, TIM-3, VISTA, CD73*, and *PD-L2* expression was significantly worse. Interestingly, *PD-L1* expression in HCC patients in the high-risk group was closely associated with EMT marker expression and prognosticates poor survival. In HCC patients, high tumor mutational burden (TMB) predicted worse patient outcomes than those with low TMB.

## Introduction

Hepatocellular carcinoma (HCC), also known as malignant hepatoma, is the most common form of primary liver malignancy and the third most common cause of cancer-related deaths worldwide (1–3). It is a multifactorial disease with viral hepatitis and excessive alcohol intake being the major risk factors globally (4). Non-alcoholic fatty liver, diabetes, aflatoxins, and immune-related conditions like autoimmune hepatitis and primary biliary cirrhosis are other common risk factors for HCC (5). HCC is predominant in patients with underlying chronic liver diseases and cirrhosis which limits treatment options for these patients (6, 7). Although surgical resection is useful in the early stages of HCC without cirrhosis recurrence continues to be a significant problem in the majority of patients (8). Liver transplantation, an alternate therapy for unresectable HCC with underlying cirrhosis, has not been very effective due to lack of compatible livers (9). Moreover, HCC is usually diagnosed at late stages such that surgical resections and liver transplantation cannot be used, leading to poor survival rate (10). Sorafenib, the systemic treatment currently approved for the treatment of advanced disease yields a sub-optimal improvement in median survival of 6.5–10.7 months in HCC patients with good liver function (11, 12). Therefore, new therapies are urgently needed for this disease.

Immunotherapy is an emerging therapeutic modality that could become a promising treatment option for HCC as, first, HCC is an inflammation-associated cancer making immunotherapy more likely to be effective (13). Second, the liver is an immune privileged organ, and thus immunotherapeutic drugs are not metabolized in the liver and have predictable pharmacokinetic profiles in cirrhotic patients (13). Third, the liver is tolerogenic to immune response to antigens that is balanced by naïve T-cell activation and further by various immunosuppressive mechanisms, including dysregulation in cytokine secretion, antigen and immune checkpoint expression, and changes in the local immune microenvironment (10, 14, 15). The clinical successes of immunotherapy in the form of immune checkpoint inhibitor (ICI) for the treatment of a number of malignancies including advanced melanoma, have opened prospects for ICIs as the potential immunotherapeutic strategy for treating HCC (16, 17).

The immune response is coordinated by a harmony between co-stimulatory and inhibitory signals (18). The activated T-cell is regulated by co-inhibitory immune checkpoint molecules, such as cytotoxic T lymphocyte-associated antigen-4 (CTLA-4), programmed cell death protein-1 (PD-1), and its ligand programmed death receptor ligand-1 (PD-L1/B7H1/CD274), all of which are responsible for maintenance of self-tolerance and prevent immune overstimulation (13, 18). The T-cell effector functions regulated by the immune checkpoint interactions are generally dysregulated or overexpressed in the tumor microenvironment leading to T-cell inhibition and downregulation of T-cell response. Thus, the blockade of immune checkpoints (co-inhibitory signals) or promotion of co-stimulatory signals can restore or amplify the antigen-specific T-cell responses for cancer therapeutics (18).

A recent phase I/II trial of nivolumab (anti-PD-1) has shown it to have an effective anticancer activity with an adequate safety profile in HCC patients (19). However, in another HCC clinical trial, the use of anti-CTLA-4 antibody in HCC resulted in more adverse events compared to anti PD-1 antibodies (20). Currently, there are several ongoing clinical trials with a small number of ICIs directed at PD-1 (nivolumab and pembrolizumab) and PD-L1 (atezolizumab) in HCC (18, 19). Given that a few genes, such as PD-1, PD-L1, and CTLA-4 enable tumors to bypass the immune system, this strategy alone may not be effective in achieving sustained clinical response in most cancer patients and further immunotherapeutic strategies are needed (21). The identification of predictive markers is of the utmost importance in this clinical setting to select a subgroup of HCC patients who are most likely to benefit from ICI therapy. Furthermore, the morphogenetic process of epithelial-to-mesenchymal transition (EMT) characterized by the acquisition of mesenchymal properties such as invasion and metastasis of tumor cells is closely linked to immune evasion of cancer cells (22, 23). Emerging evidence supports the close association of EMT status with response to multiple immune checkpoint regulators in a large number of patient tumors (24). One such report has revealed that EMT suppresses antitumor immunity through upregulation of PD-L1 in pulmonary cancer (25). However, no studies have compared the EMT markers and immune checkpoint molecule expression in HCC tumors.

With the goal of identifying prognostic immune-related molecules in HCC, we conducted a study of immune-related molecules and correlated their expression with patient prognosis in publically available HCC patient databases by deploying SurvExpress web-based platform that provides risk assessment and survival analysis in cancer datasets (26). We also assessed the relationship between the expression of immune-related molecules and EMT status of HCC cancers using this web-based tool.

## Materials and Methods

### OncoPrint Analysis of Immune Checkpoints Using cBioPortal

We used the cBioPortal’s OncoPrint^1^ across HCC patient samples to obtain a compact graphical summary of gene expression alterations in immune modulatory genes. We applied cBioPortal to study gene alterations in immune modulatory genes in Liver HCC (TCGA Provisional) case set. Genomic alterations, including copy number alterations (CNAs) (amplifications and homozygous deletions), mutations, and alterations in gene or protein expressions are summarized by glyphs and color coding. All cases are arranged as per alterations (27).

### HCC Patient Databases

We used SurvExpress, an online tool with a gene expression database of various cancer types to generate survival and risk assessment analyses of HCC patient datasets.^2^ SurvExpress provided six HCC databases, including, Hoshida Golub Liver GSE10143 with 162 patient samples, Hoshida Golub Liver GSE10186 with 118 patient samples, Tsuchiya Rusyn Liver GSE17856 with 95 patient samples, TCGA-Liver-Cancer with 422 patient samples, LIHC-TCGA-Liver HCC June 2016 with 361 patient samples, and Liver HCC TCGA database with 12 patient samples (28–30).

### Performing Risk Analysis in HCC Patients

SurvExpress utilized prognostic index (PI) or risk score, the linear part of the Cox model, to generate high-risk and low-risk groups. SurvExpress generates risk groups for risk assessment as previously described (26). Briefly, the first method splits ordered PI into two risk groups with equal number of samples equivalent to splitting the PI by the median (26). The second method uses an optimization algorithm from the ordered PI to produce risk groups (26). A log-rank test is performed along all values of the arranged PI for two groups and the split point where the *p*-value is minimum is selected by the algorithm (26). In case of more than two groups, the procedure optimizes one risk group at a time repeatedly until no changes are seen (26). The gene expression box plots of each gene and risk group are generated by SurvExpress (26).

### Validation of the Prognostic Effect of Immune Regulatory Molecules in HCC Patients

Using the SurvExpress online tool, we assessed the gene expression of 19 different immune modulators and analyzed their association with the survival of HCC patients (Cox regression analyses) in five databases (GSE10143, GSE10186, and the three TCGA datasets) with patient survival information. We also assessed the correlation of immune checkpoint molecules with recurrence-free survival in two databases (GSE10143 and TCGA-Liver-Cancer) with patient recurrence-free survival information. For HCC patients, Kaplan–Meier curves were used to estimate the survival times for overall survival and recurrence-free survival. The settings we selected for this study for duplicated genes was average of all probe sets of a gene to compute an average per sample and we used the original quantile-normalized database.

### Analysis of Tumor Mutational Burden (TMB) in HCC Patients

Data on the number of mutations per sample were obtained using cBioportal for all HCCs with available survival from the provisional TCGA data set. Tumors were classified as “high mutation burden” if they had a quantity of mutations one standard deviation above the average for the dataset. Kaplan–Meier plots were generated and log-rank tests were used to determine statistical significance.

### GeneCards Analysis for Expression of Immune Checkpoints

GeneCards is a database that provides comprehensive information on all annotated and predicted human genes (31).^3^ GeneCards online portal was used to study protein expression of immune modulators in normal hepatocytes.

### Immunohistochemistry and Pathological Evaluation

Immunohistochemistry was performed as previously described (32). Briefly, paraffin embedded tissue slides with human HCC tissue microarray (TMA) (NBP2-30221, Novus Biologicals) were deparaffinized and rehydrated, endogenous peroxidise activity was blocked with 3% hydrogen peroxide, antigen retrieval was performed in 10 mmol/L citrate buffer, and nonspecific binding was blocked with blocking reagent. HAVCR2 (ab185703, Abcam) and C10ORF54 (CL3975, Invitrogen) antibodies were applied at 1:300 and 1:20 concentrations, respectively. Slides were incubated overnight at 4°C, followed by 30 min incubation with secondary anti-mouse or rabbit antibody HRP (Dako). The chromogen used was 3-amino-9-ethylcarbazole. Human normal and cancerous lung tissue was used as the positive control for both the antibodies and a negative control, for which the primary antibodies were substituted with the same concentration of mouse or rabbit IgG. Images were captured using a Olympus CX41 microscope and QCapture software. Immunohistochemical reactivity was evaluated by two independent investigators. The expression of HAVCR2 and C10ORF54 were categorized into positive staining or no staining.

### Statistical Analysis

For risk assessment generated by SurvExpress, a *p*-value of the difference in expression among risk groups is obtained from a Student’s *t*-test for two risk groups. A log-rank test was used to produce the concordance index and the *p*-value testing for equality of survival curves for survival analysis using SurvExpres, and the correlation coefficient estimated from deviance residuals (33). In addition, an estimation of the hazard ratio (HR) between the groups is generated. This is estimated by another Cox model using the risk group prediction as the covariate.

## Results

### The Alterations in Immune Modulatory Genes in HCC

To identify immune modulatory molecules involved in immune escape in HCC, we assessed a panel of 19 genes based on previous studies on immune modulatory genes linked with overall survival and progression-free survival in different cancers. These included those associated with immune stimulatory genes, such as *CD80, CD28, CD27, GITR* (*TNFRSF18*), *Galectin*-9 (*LGALS9*), *CD137* (*TNFRSF9*), *FASLG*, and immune inhibitory genes, such as *TIM3* (*HAVCR2*), *B7-H4* (*VTCN1*), *B7-H3* (*CD276*), B and T lymphocyte attenuator (*BTLA*), *HVEM* (*TNFRSF14*), *PD-L1* (*CD274*), *PD-L2* (*PDCD1LG2*), *LAG-3, VISTA* (*C10ORF54*), *CD73* (*NT5E*), *IDO-1, TIGIT*.

**Figure 1:**
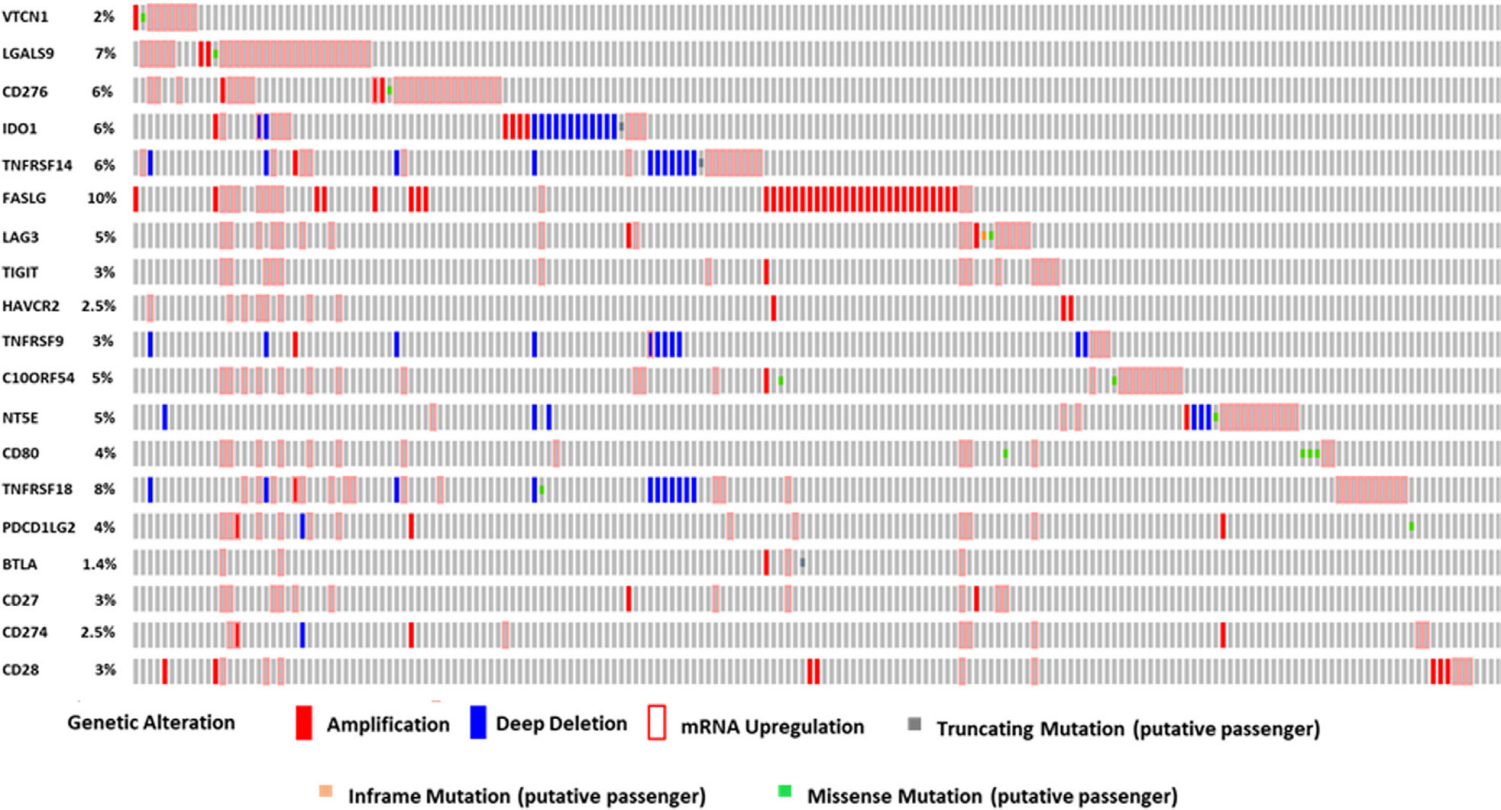
The OncoPrint from a query for alterations in expression of immune modulator genes in HCC patients. Rows and columns represents genes and patients, respectively. Genomic alterations, including CNAs (homozygous deletions and amplifications), mutations, and variation in gene or protein expression are summarized by glyphs and color coding. The cases are sorted as per alterations.

We performed OncoPrint analysis using cBioPortal to interrogate the expression profiles and any possible genetic alterations for these immune modulatory molecules in tumors of HCC patients (*n* = 440). An OncoPrint is a concise and compact graphical summary of genomic alterations in multiple genes across a set of tumor samples. From the OncoPrint, of the 440 HCC cases, amplification and mRNA upregulation were identified in *FASLG, TIGIT, HAVCR2, CD27*, and *CD28* in 45 cases (10%), 15 cases (3%), 11 cases (2.5%), 13 cases (3%), and 15 cases (3%), respectively (Figure 1). Amplification, deep deletion, and mRNA upregulation were identified in *TNFRSF9* and *CD274* in 15 cases (3%) and 11 cases (2.5%), respectively. In Figure 1, amplification, mRNA upregulation and missense mutation were noted in 24 cases (5%) for *C10ORF54*, 9 cases (2%) for *VTCN1*, 29 cases (7%) for *LGALS9*, and 26 cases (6%) for *CD276*. Amplification, deep deletion, mRNA upregulation, and missense mutation were identified in *NT5E* [22 cases (5%)], *TNFRSF18* [35 cases (8%)], and *PD-L2* [16 cases (4%)]. Amplification, deep deletion, mRNA upregulation, and truncating mutation were noted in *IDO1* [27 cases (6%)] and *TNFRSF14* [27 cases (6%)]. Furthermore, amplification, mRNA upregulation, inframe mutation, and missense mutation were observed in *LAG3* [20 cases (5%)]. Amplification, mRNA upregulation, and truncating mutation were identified in *BTLA* in 6 cases (1.4%). While 17 cases (4%) for *CD80* showed both mRNA upregulation and missense mutation (Figure 1).

### Immune Modulatory Genes Are Aberrantly Expressed in Human HCC Tumors

Using SurvExpress we examined transcriptome profiling studies to produce high-risk versus low-risk HCC signatures. Based on transcriptome profiles of the TCGA-Liver-Cancer patient dataset, the clustering analysis differentiated a total of 422 HCC patient samples into high-risk and low-risk groups. Box plot was generated in the results of SurvExpress, where the gene expression per gene is plotted along its risk groups. This plot is useful to visualize differences in gene expression values between high and low-risk groups.

**Figure 2:**
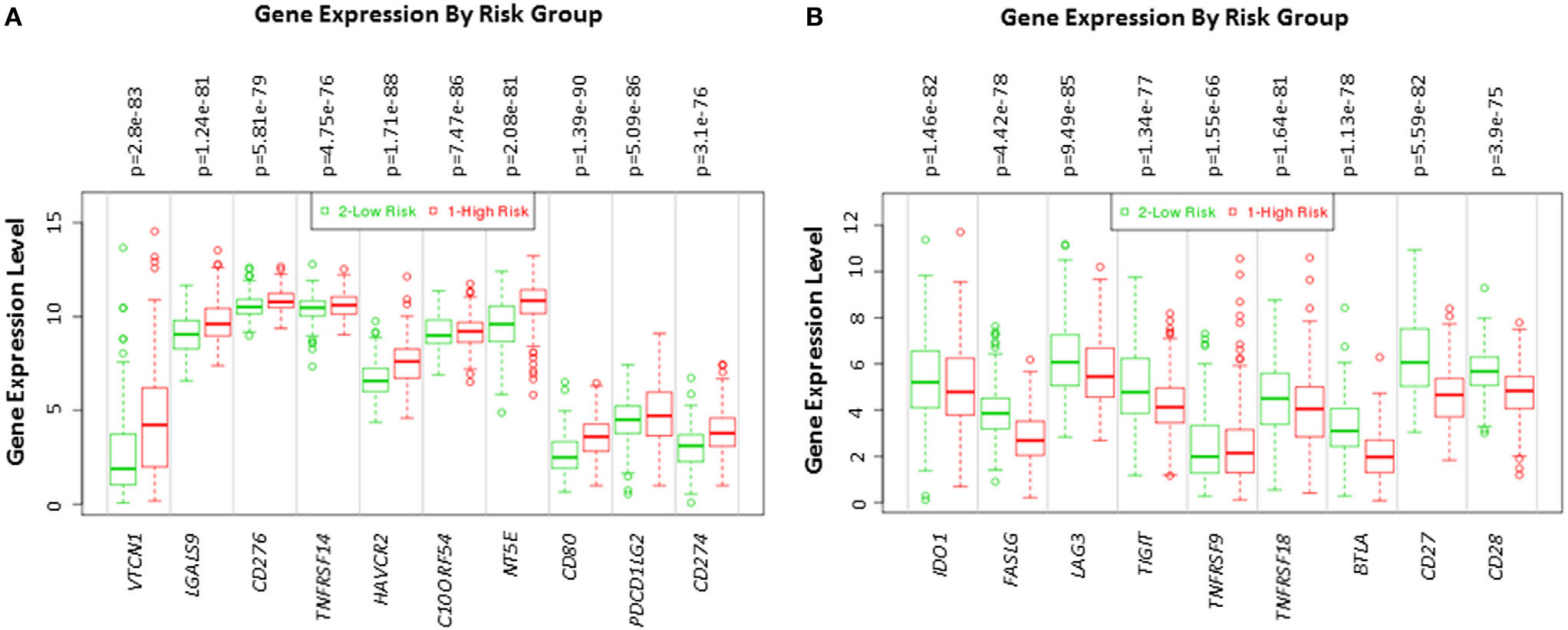
Gene expression of immune modulators in HCC patients based on risk group. Box plot of gene expression of **(A)** immune modulators that statistically correlate with high-risk prognostic score and **(B)** immune modulators that statistically correlates with low-risk prognostic score in 422 HCC patients from the TCGA-Liver Cancer dataset. Risk assessed is risk of reduced survival. Red box represents high-risk group and green box represents low-risk group. Each gene is shown on the *x*-axis. *X*-axis also shows a *p*-value of the expression difference between the two risk groups. The expression levels are shown on the *y*-axis.

The expression of *VTCN1, LGALS9, CD276, TNFRSF14, HAVCR2, C10ORF54, NT5E, CD80, PDCD1LG2*, and *CD274* genes statistically significantly correlates with high-risk signature (*p* < 0.05) (Figure 2A). Immune-related genes *IDO-1, FASLG, LAG-3, TIGIT, TNFRSF9, TNFRSF18, BTLA, CD27*, and *CD28* expression significantly correlates with low-risk signatures (*p* < 0.05) (Figure 2B). Risk assessed in this study was reduced survival. Risk assessment of high versus low-risk for all six HCC patient datasets are depicted in Table 1.

**Table 1:** Risk assessment of high versus low-risk.

High expression in high-risk group	Low expression in high-risk group
*B7-H4*	*IDO-1*
*LGALS9*	*FASLG*
*B7-H3*	*LAG-3*
*TNFRSF14*	*TIGIT*
*TIM-3*	*TNFRSF9*
*VISTA*	*TNFRSF18*
*NT5E*	*BTLA*
*CD80*	*CD27*
*PD-L2*	*CD28*
*PD-L1*	

### Immune Biomarkers Prognosticates Clinical Outcome in HCC Patients

**Figure 3:**
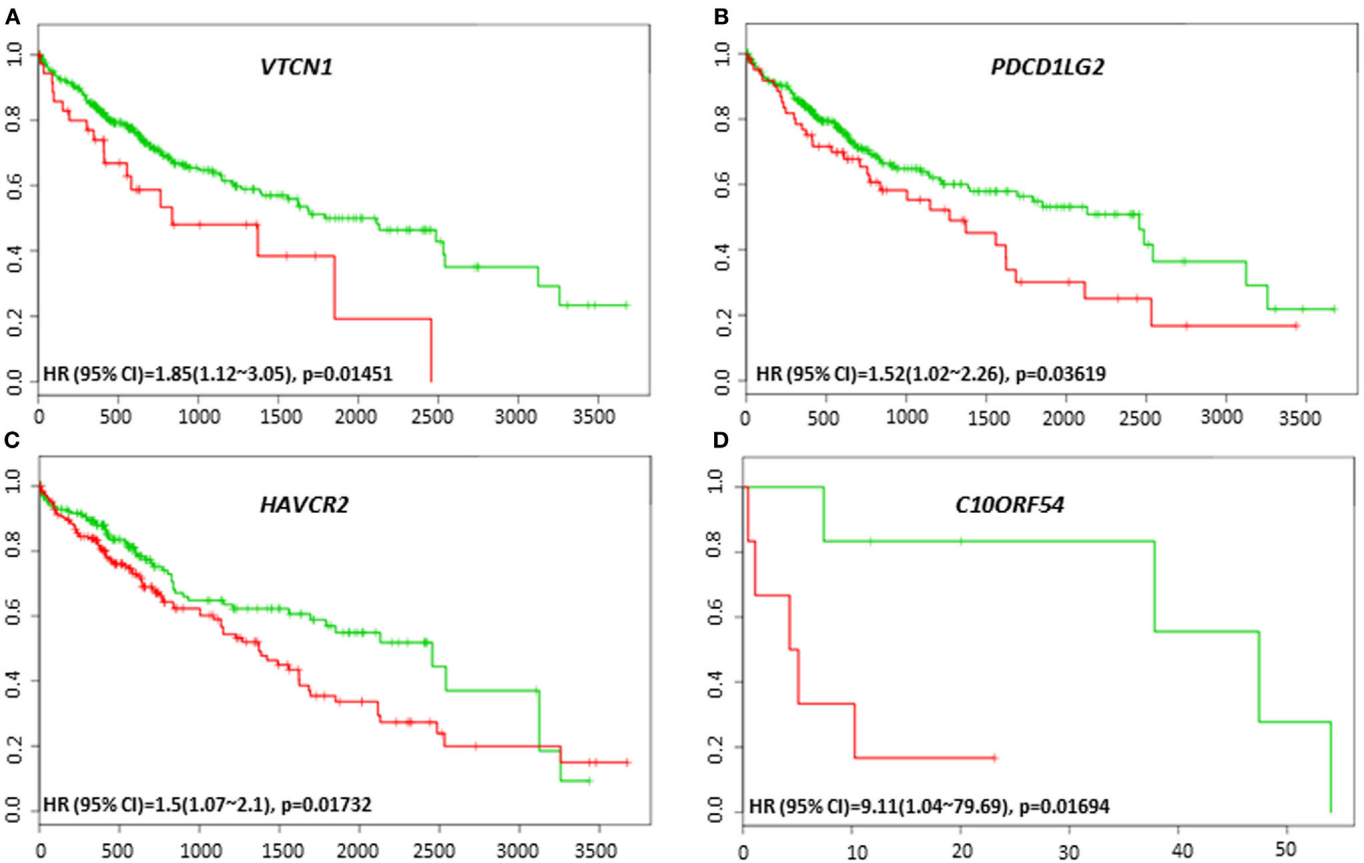
Relationship of immune modulators and survival in HCC patients. Kaplan–Meier survival curves from SurvExpress for the analysis of survival and gene expression of **(A)** *VTCN1*, **(B)** *PDCD1LG2*, **(C)** *HAVCR2*, and **(D)** *C10ORF54* in HCC patient samples. Green curve represents low-risk group while red curve represents high-risk group. The study time (months) is presented in the *x*-axis. The insert shows the hazard ratio, confidence interval, and Log-Rank Equal Curves *p* value. Markers (+) represent censoring samples.

The lack of robust predictive biomarkers to monitor HCC patients at high risk for poor prognosis has been a major obstacle in the clinics. To investigate whether the immune-related genes have prognostic and predictive value in HCC, we utilized six different HCC datasets within SurvExpress to examine the overall survival and recurrence-free survival in HCC patients. Kaplan–Meier survival risk curves for the different immune genes were generated. Notably, altered expression of *VTCN1* [HR: 1.85, 95% confidence interval (CI): 1.12~3.05, Log-Rank Equal Curves *p* = 0.01451] and *PDCD1LG2* (HR: 1.52, 95% CI: 1.02~2.26, Log-Rank Equal Curves *p* = 0.03619) in the TCGA HCC 361 patient cohort was associated with worse overall survival (Figures 3A,B). In the TCGA Liver Cancer 422 patient cohort, *HAVCR2* (HR: 1.5, 95% CI: 1.07~2.1, Log-Rank Equal Curves *p* = 0.01732) expression in high-risk group correlated with low overall survival (Figure 3C). In TCGA 12 HCC patients, *C10ORF54* expression correlated with worse survival (HR: 9.11, CI = 1.04~79.69, *p* = 0.01694) (Figure 3D).

**Figure 4:**
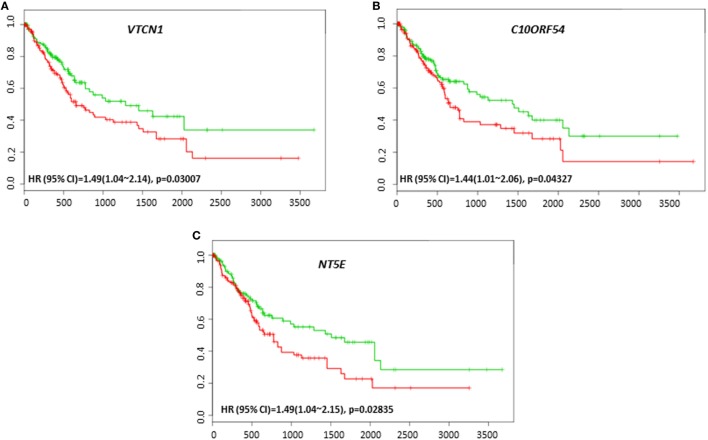
Relationship of immune modulators and recurrence-free survival in HCC patients. Kaplan–Meier curves produced using the SurvExpress for the analysis of recurrence-free survival and gene expression of **(A)** *VTCN1*, **(B)** *C10ORF54*, and **(C)** *NT5E* in HCC patient samples. Green curve represents low-risk group, while red curve represents high-risk group. The study time (months) is presented in the *x*-axis. The insert shows the hazard ratio, confidence interval, and Log-Rank Equal Curves *p* value. Markers (+) represent censoring samples.

To investigate the possible roles of immune genes in HCC relapse, we assessed the relationships between their gene expression level and recurrence-free survival using SurvExpress. We observed that *VTCN1* expression, which correlated with poor survival was also associated with poor recurrence-free survival in the cohort of TCGA 422 patients (HR: 1.49, CI: 1.04~2.14, Log-Rank Equal Curves *p* = 0.03007) (Figure 4A). *C10ORF54* expression also correlated with low recurrence-free survival in the same cohort of 422 patients (HR: 1.44, CI: 1.01~2.06, Log-Rank Equal Curves *p* = 0.04327) (Figure 4B). This cohort also showed that *NT5E* expression correlated with poor recurrence-free survival (HR: 1.49, CI: 1.04~2.15, Log-Rank Equal Curves *p* = 0.02835) (Figure 4C).

### Coordinate Expression of PD-L1 (CD274), PD-1, and CTLA-4 and Immune Modulatory Genes in HCC

**Figure 5:**
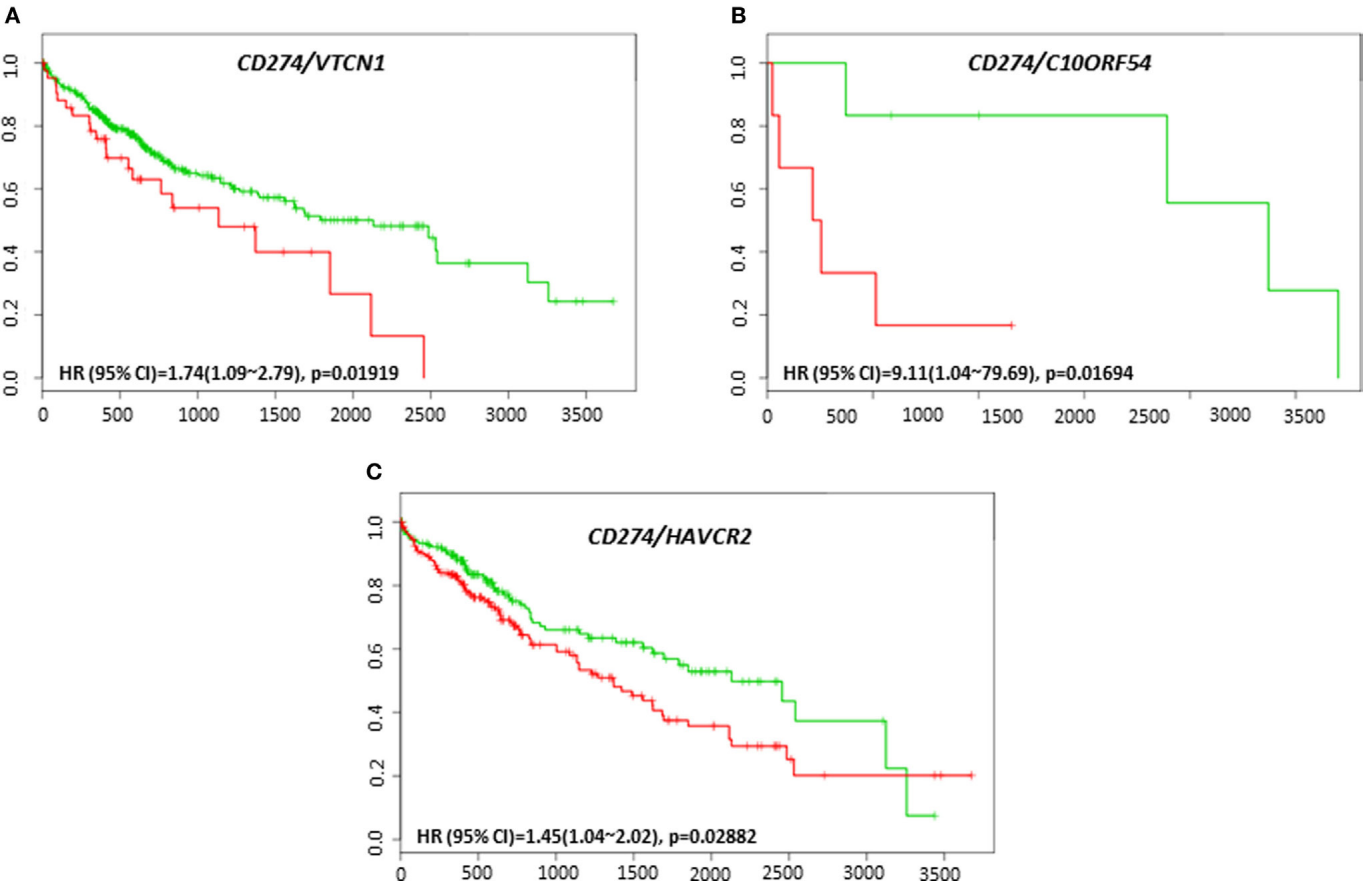
Relationship of immune modulators in combination with PD-L1 (CD274) and survival in HCC patients. Kaplan–Meier survival curves generated using the SurvExpress for the analysis of survival and gene expression of **(A)** *CD274/VTCN1*, **(B)** *CD274/C10ORF54*, and **(C)** *CD274/HAVCR2* in HCC patient samples. Green curve represents low-risk group, while red curve represents high-risk group. The study time (months) is presented in the *x*-axis. The insert shows the hazard ratio, confidence interval, and Log-Rank Equal Curves *p* value. Markers (+) represent censoring samples.

**Figure 6:**
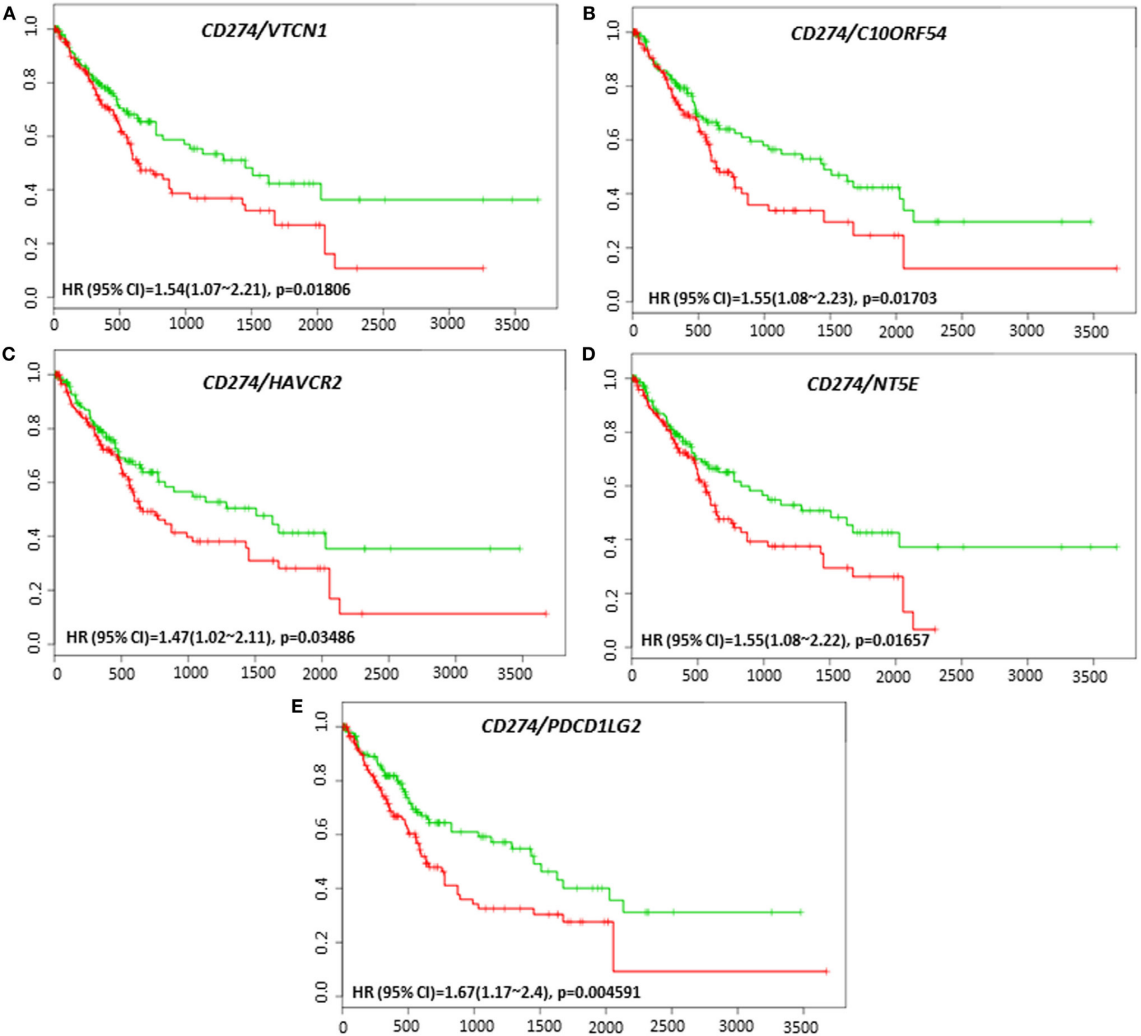
Relationship of immune modulators in combination with PD-L1 (CD274) and recurrence-free survival in HCC patients. Kaplan–Meier curves produced using the SurvExpress for the analysis of recurrence-free survival and gene expression of **(A)** *CD274/VTCN1*, **(B)** *CD274/C10ORF54*, **(C)** *CD274/HAVCR2*, **(D)** *CD274/NT5E*, and **(E)** *CD274/PDCD1LG2* in HCC patient samples. Green curve represents low-risk group, while red curve represents high-risk group. The study time (months) is presented in the *x*-axis. The insert shows the hazard ratio, confidence interval, and Log-Rank Equal Curves *p* value. Markers (+) represent censoring samples.

The clinical response to anti-PD-L1, anti-PD-1, or anti-CTLA-4 targeted therapies can vary in different tumor types, and much effort has been directed toward finding predictive biomarkers to help identify patients who will derive the most benefit from these therapies. In HCC, the coordinated expression of other immune regulators with PD-L1, PD-1, and CTLA-4 in tumor tissue have been less well-studied. The overall survival and recurrence-free survival of immune modulators were analyzed in combination with PD-L1, PD-1, and CTLA-4 to assess any additional benefit through the combination. *PD-L1, PD-1*, or *CTLA-4* gene expression alone did not show poor survival in HCC patient datasets. However, coordinate expression of *VTCN1* (HR: 1.74, CI: 1.09~2.79, Log-Rank Equal Curves *p* = 0.01919), *C10ORF54* (HR: 9.11, CI: 1.04~79.69, Log-Rank Equal Curves *p* = 0.01694), and *HAVCR2* (HR: 1.45, CI: 1.04~2.02, Log-Rank Equal Curves *p* = 0.02882) showed significant overall worse survival when combined with *PD-L1* (CD274) (Figures 5A–C). *VTCN1* (HR: 1.54, CI: 1.07~2.21, Log-Rank Equal Curves *p* = 0.01806), *C10ORF54* (HR: 1.55, CI: 1.08~2.23, Log-Rank Equal Curves *p* = 0.01703), *HAVCR2* (HR: 1.47, CI: 1.02~2.11, Log-Rank Equal Curves *p* = 0.03486), *NT5E* (HR:1.55, CI: 1.08~2.22, Log-Rank Equal Curves *p* = 0.01657), and *PDCD1LG2* (HR: 1.67, CI: 1.17~2.4, Log-Rank Equal Curves *p* = 0.004591) showed significant recurrence-free survival benefit when combined with *PD-L1* (Figures 6A–E).

**Figure 7:**
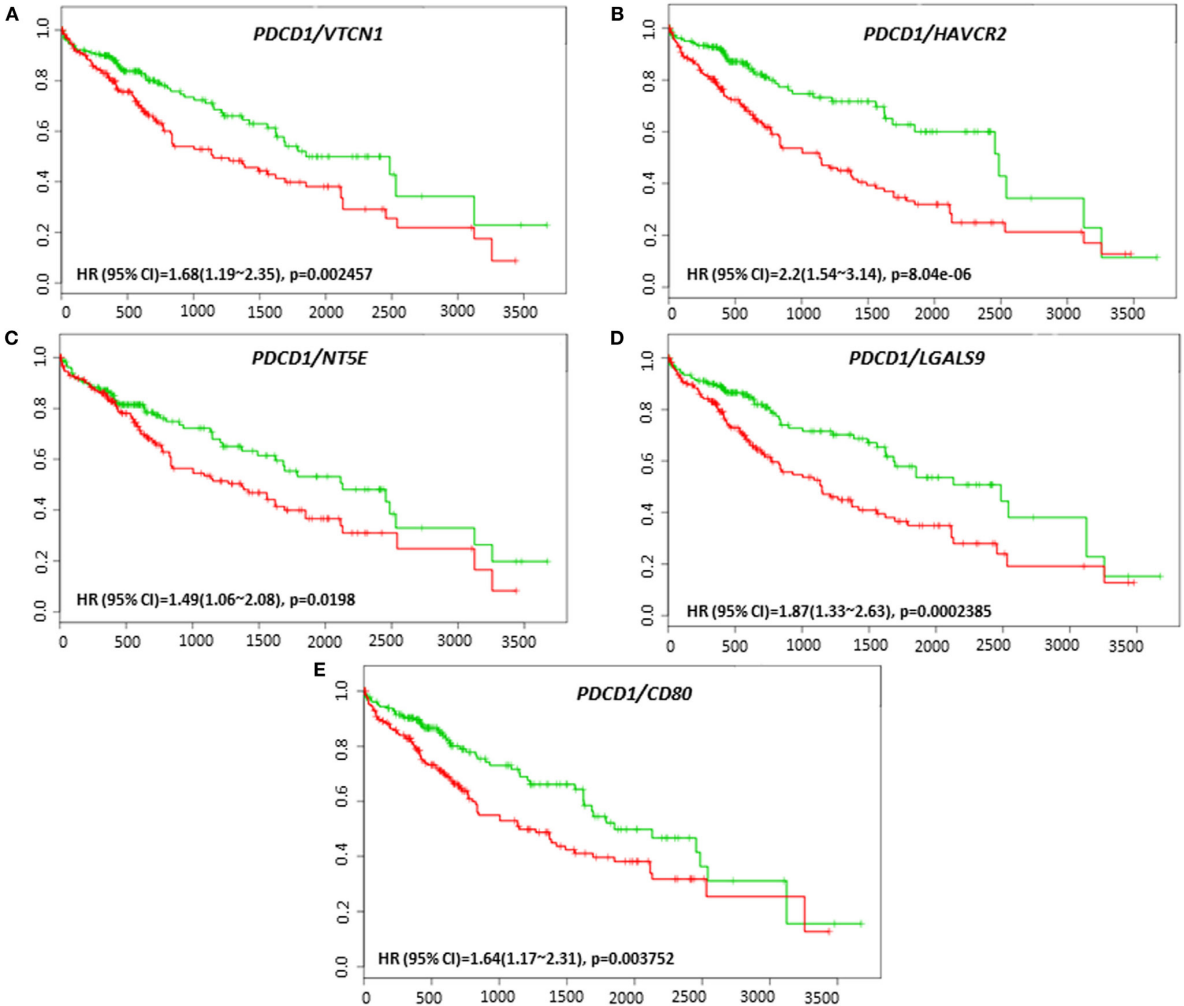
Relationship of immune modulators in combination with PDCD1 and survival in HCC patients. Kaplan–Meier survival curves generated using the SurvExpress for the analysis of survival and gene expression of **(A)** *PDCD1/VTCN1*, **(B)** *PDCD1/HAVCR2*, **(C)** *PDCD1/NT5E*, **(D)** *PDCD1/LGALS9*, and **(E)** *PDCD1/CD80* in HCC patient samples. Green curve represents low-risk group, while red curve represents high-risk group. The study time (months) is presented on the *x*-axis. The insert shows the hazard ratio, confidence interval, and Log-Rank Equal Curves *p* value. Markers (+) represent censoring samples.

**Figure 8:**
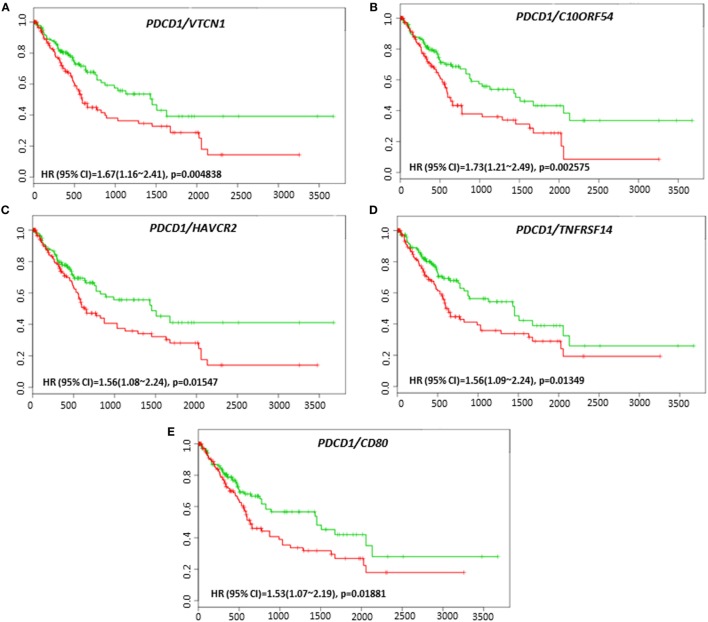
Relationship of immune modulators in combination with PDCD1 and recurrence-free survival in HCC patients. Kaplan–Meier curves produced using the SurvExpress for the analysis of recurrence-free survival and gene expression of **(A)** *PDCD1/VTCN1*, **(B)** *PDCD1/C10ORF54*, **(C)** *PDCD1/HAVCR2*, **(D)** *PDCD1/TNFRSF14*, and **(E)** *PDCD1/CD80* in HCC patient samples. Green curve represents low-risk group, while red curve represents high-risk group. The study time (months) is presented on the *x*-axis. The insert shows the hazard ratio, confidence interval, and Log-Rank Equal Curves *p* value. Markers (+) represent censoring samples.

Coordinate expression of *VTCN1* (HR: 1.68, CI: 1.19~2.35, Log-Rank Equal Curves *p* = 0.002457), *HAVCR2* (HR: 2.2, CI: 1.54~3.14, Log-Rank Equal Curves *p* = 8.04E-06), *NT5E* (HR: 1.49, CI: 1.06~2.08, Log-Rank Equal Curves *p* = 0.0198), *LGALS9* (HR: 1.87, CI: 1.33~2.63, Log-Rank Equal Curves *p* = 0.0002385), and *CD80* (HR: 1.64, CI: 1.17~2.31, Log-Rank Equal Curves *p* = 0.003752) showed significant overall worse survival when combined with *PD-1 (PDCD1)* (Figures 7A–E). In combination with *PD-1, VTCN1* (HR: 1.67, CI: 1.16~2.41, Log-Rank Equal Curves *p* = 0.004838), *C10ORF54* (HR: 1.73, CI: 1.21~2.49, Log-Rank Equal Curves *p* = 0.002575), *HAVCR2* (HR: 1.56, CI: 1.08~2.24, Log-Rank Equal Curves *p* = 0.01547), *TNFRSF14* (HR:1.56, CI: 1.09~2.24, Log-Rank Equal Curves *p* = 0.01349), and *CD80* (HR: 1.53, CI: 1.07~2.19, Log-Rank Equal Curves *p* = 0.01881) showed significant recurrence-free survival (Figures 8A–E).

**Figure 9:**
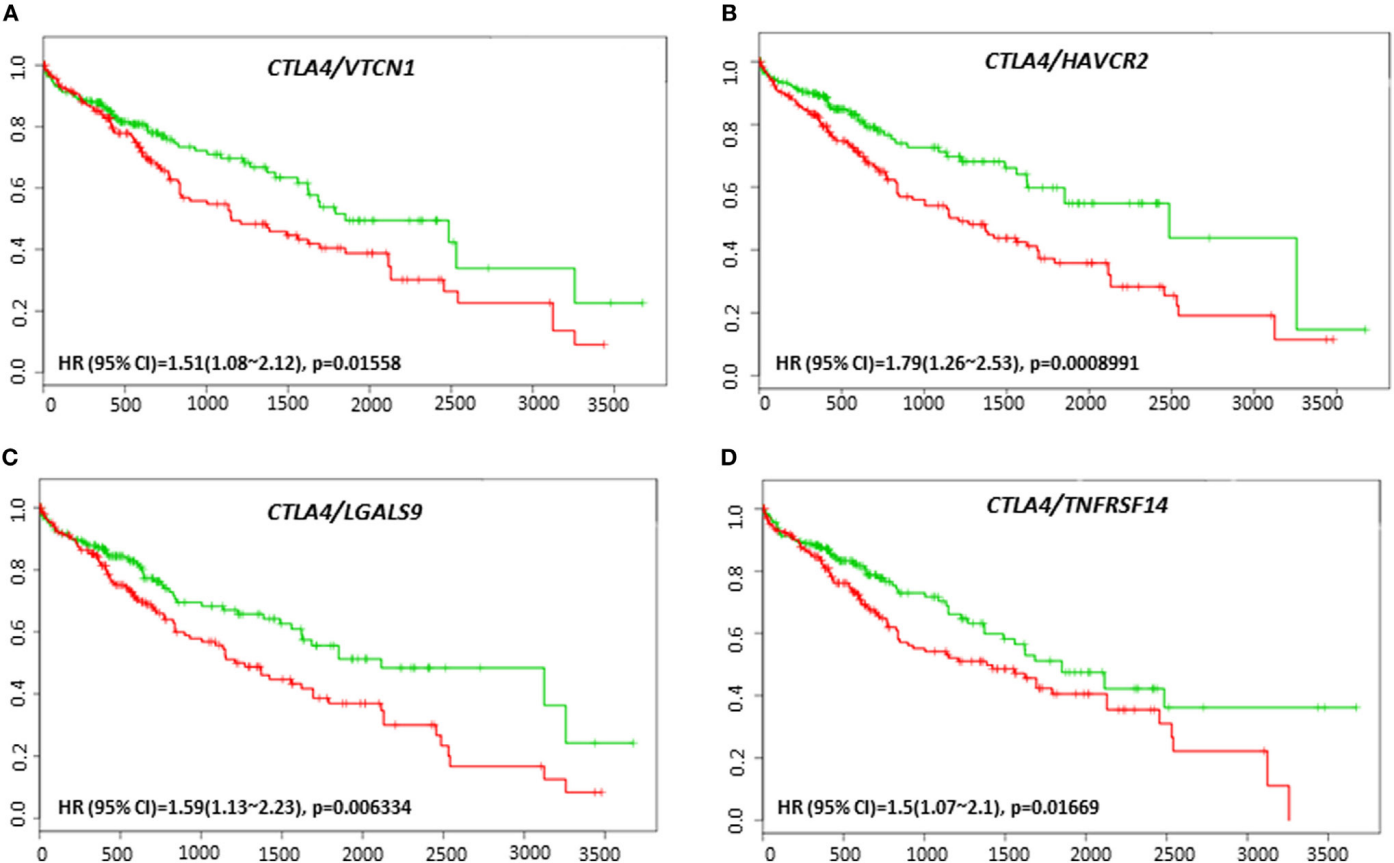
Relationship of immune modulators in combination with CTLA-4 and survival in HCC patients. Kaplan–Meier survival curves generated using the SurvExpress for the analysis of survival and gene expression of **(A)** *CTLA-4/VTCN1*, **(B)** *CTLA-4/HAVCR2*, **(C)** *CTLA-4/LGALS9*, and **(D)** *CTLA-4/TNFRSF14* in HCC patient samples. Green curve represents low-risk group, while red curve represents high-risk group. The study time (months) is presented on the *x*-axis. The insert shows the hazard ratio, confidence interval, and Log-Rank Equal Curves *p* value. Markers (+) represent censoring samples.

**Figure 10:**
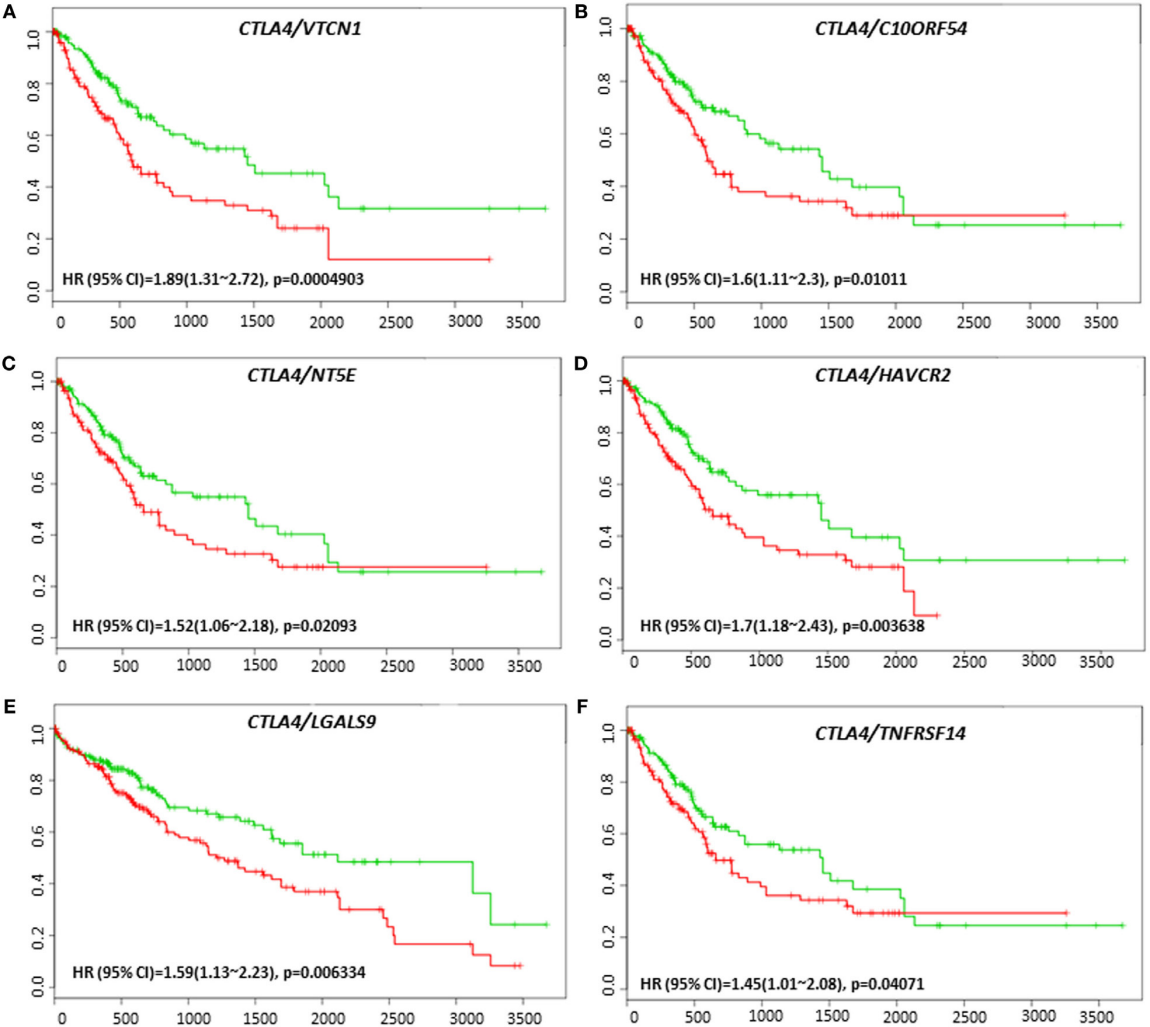
Relationship of immune modulators in combination with CTLA-4 and recurrence-free survival in HCC patients. Kaplan–Meier curves produced using the SurvExpress for the analysis of recurrence-free survival and gene expression of **(A)** *CTLA-4/VTCN1*, **(B)** *CTLA-4/C10ORF54*, **(C)** *CTLA-4/NT5E*, **(D)** *CTLA-4/HAVCR2*, **(E)** *CTLA-4/LGALS9*, and **(F)** *CTLA-4/TNFRSF14* in HCC patient samples. Green curve represents low-risk group, while red curve represents high-risk group. The study time (months) is presented on the *x*-axis. The insert shows the hazard ratio, confidence interval, and Log-Rank Equal Curves *p* value. Markers (+) represent censoring samples.

*VTCN1* (HR: 1.51, CI: 1.08~2.12, Log-Rank Equal Curves *p* = 0.01558), *HAVCR2* (HR: 1.79, CI: 1.26~2.53, Log-Rank Equal Curves *p* = 0.0008991), *LGALS9* (HR: 1.59, CI: 1.13~2.23, Log-Rank Equal Curves *p* = 0.006334), and *TNFRSF14* (HR: 1.5, CI: 1.07~2.1, Log-Rank Equal Curves *p* = 0.01669) showed significant overall worse survival when combined with *CTLA-4* (Figures 9A–D). Coordinate expression of *CTLA-4* with *VTCN1* (HR: 1.89, CI: 1.31~2.72, Log-Rank Equal Curves *p* = 0.0004903), *C10ORF54* (HR: 1.6, CI: 1.11~2.3, Log-Rank Equal Curves *p* = 0.01011), *NT5E* (HR: 1.52, CI: 1.06~2.18, Log-Rank Equal Curves *p* = 0.02093), *HAVCR2* (HR:1.7, CI: 1.18~2.43, Log-Rank Equal Curves *p* = 0.003638), *LGALS9* (HR: 1.59, CI: 1.13~2.23, Log-Rank Equal Curves *p* = 0.006334), and *TNFRSF14* (HR: 1.45, CI: 1.01~2.08, Log-Rank Equal Curves *p* = 0.04071) showed significant recurrence-free survival (Figures 10A–F).

### HAVCR2 and C10ORF54 Is Expressed in HCC Patient Tumors

**Figure 11:**
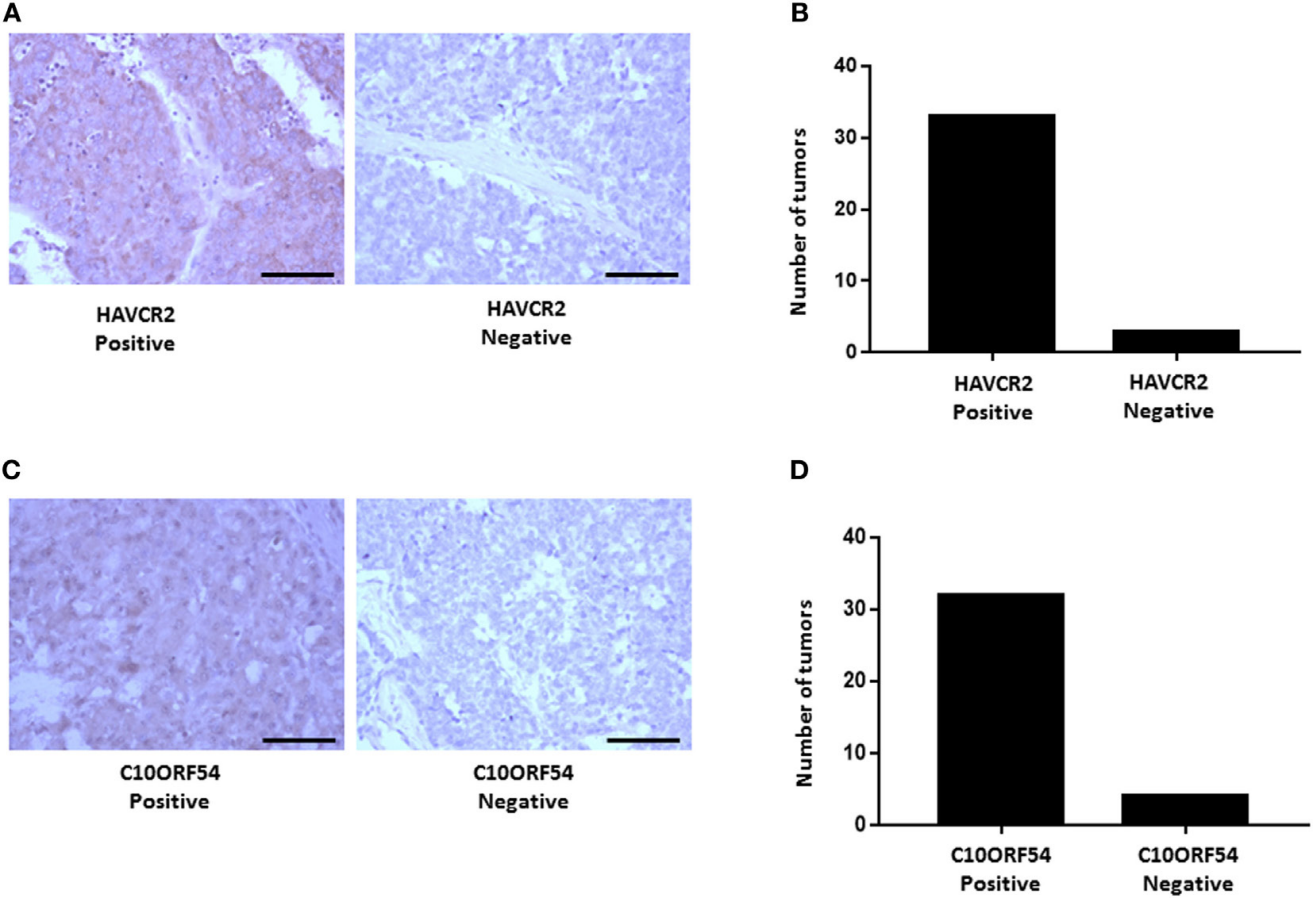
HAVCR2 and C10ORF54 immuno staining in HCC tumor tissue. **(A)** Localization of HAVCR2 in HCC tumor biopsies. **(B)** Graph represents number of tumors scored as HAVCR2 positive or negative. **(C)** Localization of C10ORF54 in HCC tumor biopsies. **(D)** Graph represents number of tumors scored as C10ORF54 positive or negative. Scale bar indicates 20× magnification.

We next validated the protein expression of HAVCR2 and C10ORF54, two immune markers associated with poor survival in HCC patients in combination with either PD-L1, PD-1, or CTLA-4. Protein expression patterns in HCC tumors were determined by immunohistochemical staining of a TMA comprising of tumors from 36 patients with stage I, II, III, IIIB, IVA, and IVB HCC. HAVCR2 expression was detected in 88% of HCC patient tumors (Figures 11A,B). The subcellular location was identified as predominantly cytoplasmic and membranous. C10ORF54 expression was detected in 91% of HCC patient tumors (Figures 11C,D). The subcellular location was identified as predominantly cytoplasmic.

### Expression of PD-L1 in HCC Tumors Is Correlated With an EMT Phenotype

**Figure 12:**
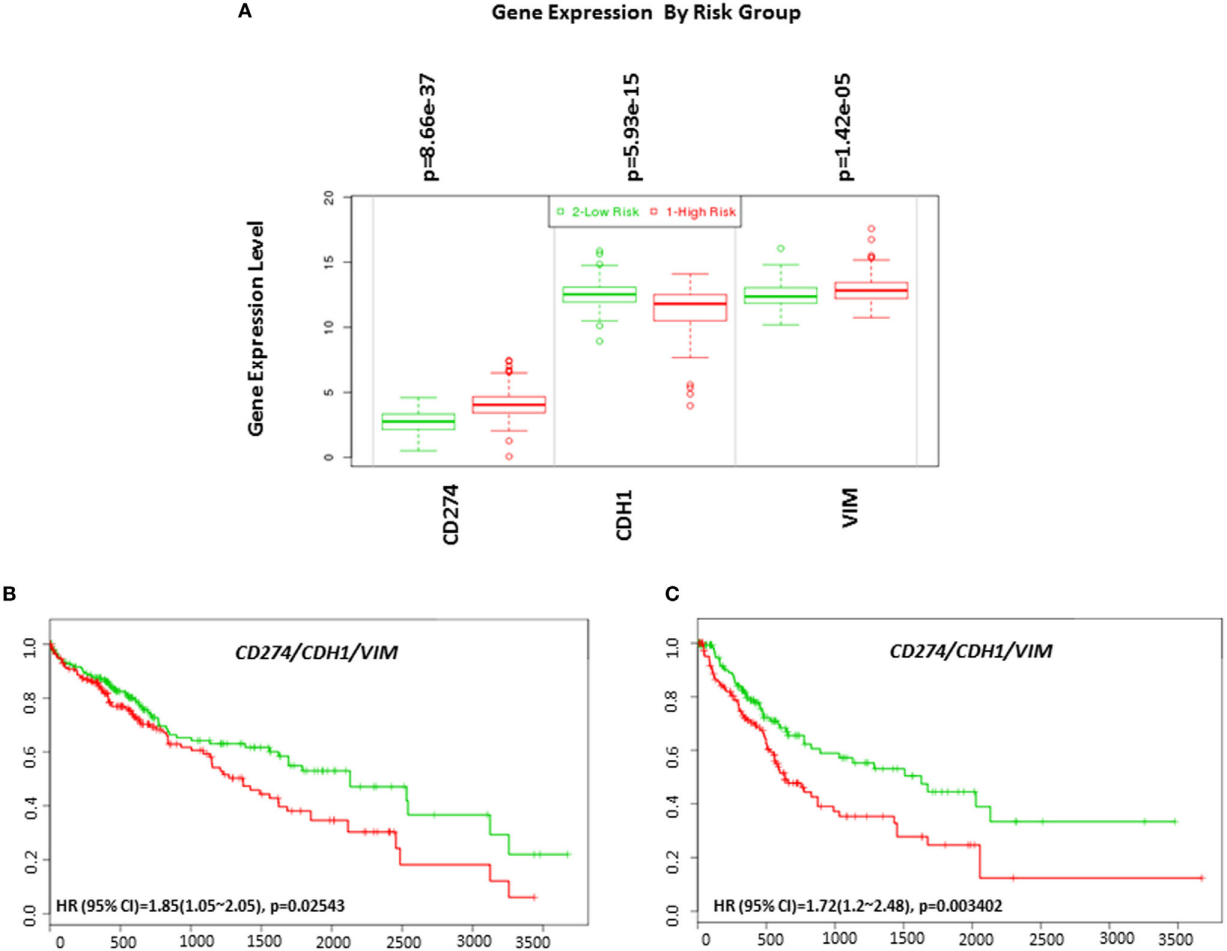
Gene expression of EMT markers in HCC based on risked group and their relationship in combination with programmed death receptor ligand-1 (CD274) and overall survival and recurrence-free survival in HCC patients. **(A)** Box plot of gene expression of EMT markers that statistically correlates with high-risk prognostic score in 422 HCC patients from the TCGA-Liver Cancer dataset. Risk assessed is risk of reduced survival. Red box represents high-risk group and green box represents low-risk group. Each gene is shown on the *x*-axis. *X*-axis also shows a *p*-value of the expression difference between the two risk groups. The expression levels are shown on the *y*-axis. Kaplan–Meier curves produced using the SurvExpress for the analysis of **(B)** overall survival and gene expression of CD274/CDH1/VIM and **(C)** recurrence-free survival and gene expression of CD274/CDH1/VIM in HCC patient samples. Green curve represents low-risk group, while red curve represents high-risk group. The study time (months) is presented in the *x*-axis. The insert shows the hazard ratio, confidence interval, and Log-Rank Equal Curves *p* value. Markers (+) represent censoring samples.

EMT is an important biological process involved in the progression and immune evasion of cancers. In HCC, EMT contributes to a poor prognosis (34, 35). Emerging research has found higher expression of PD-L1 in mesenchymal cells in non-small cell lung carcinoma (36). Therefore, we examined the relationship between the EMT phenotype and PD-L1 expression in HCC. By analyzing risk assessment using the TCGA-Liver-Cancer patient dataset (422 HCC patient samples) we confirmed that high expression of *PD-L1* and mesenchymal marker *VIM* and low expression of epithelial marker *CDH1* genes significantly associated with a high-risk signature (*p* < 0.05) (Figure 12A).

Although *PD-L1* gene expression alone did not significantly correlate with poor survival in HCC patient datasets, coordinate expression of *CDH1* and *VIM* showed worse overall survival (HR: 1.85, CI: 1.05~2.05, Log-Rank Equal Curves *p* = 0.02543) and recurrence-free survival (HR: 1.72, CI: 1.2~2.48, Log-Rank Equal Curves *p* = 0.003402) when combined with *PD-L1* (Figures 12B,C). This study shows that high expression of PD-L1 in HCC patients is associated with an EMT phenotype.

### Protein Expression in Normal Hepatocytes

GeneCards online portal was utilized to select tumor-associated immune regulatory genes with minimal or no expression in normal tissue and overexpression in HCC tumor cells. GeneCards online portal was used to study protein expression of immune modulators in normal hepatocytes (Table 2). The majority of immune modulators are not expressed in normal hepatocytes. Some of the immune modulators including Galectin-9 and B7H3 have low protein expression in normal hepatocytes, while *LAG-3* and *CD73* showed low mRNA expression in normal hepatocytes. This data indicate that these biomarkers may be specifically expressed in HCC tumors and not in normal healthy cells but may be targeted safely.

**Table 2:** Estimated protein expression in normal hepatocytes.

Immune modulator	Estimated protein expression log_10_ (ppm) in liver
B7-H4	No expression
LGALS9	Low expression
B7-H3	Low expression
IDO-1	No expression
HVEM	No expression
FASLG	No expression
LAG3	Low mRNA expression
TIGIT	No expression
TIM-3	No expression
CD137	No data
VISTA	Low expression
CD73	Low mRNA expression
CD80	No expression
GITR	No expression
PD-L2	No expression
BTLA	No expression
CD27	No expression
PD-L1	No expression
CD28	No expression

### TMB in HCC Patients

**Figure 13:**
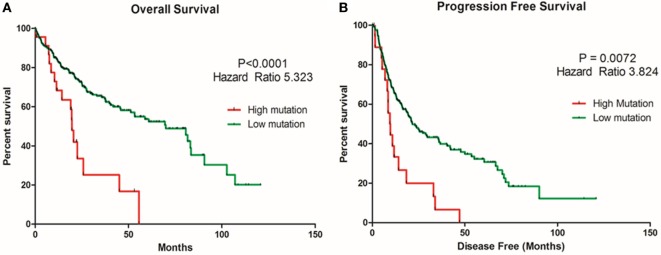
Relationship of tumor mutational burden (TMB) and overall and progression-free survival in HCC patients. Kaplan–Meier curves produced using the cBioportal for the analysis of **(A)** overall survival and **(B)** progression-free survival in HCC patient samples. Green curve represents low mutation or TMB, while red curve represents high mutation or TMB. The study time (months) is presented on the *x*-axis.

TMB or mutation load is the total number of mutations present in a tumor specimen. TMB is emerging as a reliable biomarker for predicting sensitivity to ICIs as immune checkpoint marker testing alone has proven insufficient in many cancers for patient selection (37). In non-small cell lung cancer and melanoma, high TMB has been associated with a higher likelihood of tumor responsiveness to treatment with PD-1 or PD-L1 immunotherapy strategies (38, 39). However, the value of TMB as an objective biomarker in the setting of HCC has not been explored. We sought to determine whether TMB could be associated with overall survival and progression-free survival in HCC patients. Patients with a high TMB had significantly poor overall survival and progression-free survival than those with a lower TMB (Figures 13A,B). As TMB-high cancers are likely to harbor neoantigens, making them targets of immune cells, utilizing TMB as a biomarker may help select HCC patients for ICI blockade therapy.

## Discussion

Implementation of immune regulatory drugs such as ICIs has elicited a remarkable clinical response and is becoming the new foundation for treatment of various malignancies. Currently, immunotherapy in the form of ICI may represent an effective treatment modality for HCC, mainly for advanced and recurrent HCC where no other effective options are available. This study identified many immune regulatory genes that were aberrantly expressed in HCC patient tumors. Immune regulatory genes *VTCN1, PDCD1LG2, HAVCR2*, and *C10ORF54* were associated with overall poor survival and *VTCN1, C10ORF54*, and *NT5E* predicted recurrence-free survival in HCC patients. *VTCN1, C10ORF54, HAVCR2, NT5E*, and *PDCDLG2* in combination with PD-L1 functioned as robust markers that could prognosticate poor prognosis in these patients.

Identifying robust predictive immune biomarkers as useful tools to monitor patients at high risk for poor prognosis and to predict response to the ICI in patients is becoming popular by study of tumor immune microenvironment. For instance, PD-L1 expression in tumors has been shown to be a predictive biomarker for poor prognosis and is also utilized as an important biomarker to predict the response to anti-PD-1 antibodies (40, 41). These findings support the relevance of immune regulatory molecules as biomarkers in the clinics. Given that only a subset of patients express PD-L1, and the majority of patients fail to demonstrate durable response and expression level of PD-L1 can fluctuate throughout the course of treatment; identifying other immune biomarkers could play an important role to further improve patient outcome. Based on immune biomarker expression, therapies will need to be employed on an individualized basis to ensure the best possible responses.

We found the negative regulator of T-cell response, V-set domain-containing T-cell activation inhibitor 1, VTCN1, (also named as B7-H4, B7S1, or B7x) was aberrantly expressed in HCC patients in the high-risk group and B7-H4 positivity was a statistically significant predictor of poor overall survival and recurrence-free survival. Studies have confirmed the high expression of B7-H4 in a variety of human tumors, including HCC (42, 43). In another study, soluble B7-H4 detected in blood samples from HCC patients was closely associated with advanced tumor stage, poor overall survival, and higher recurrence rate (44, 45). However, the function of B7-H4 in HCC tumors remains unknown. B7-H4 has been previously proposed to function as a ligand for BTLA (also known as CD272), an Ig superfamily member. The B7-H4–Ig fusion protein inhibits T-cell activation (46).

The inhibitory immune checkpoint molecule, V-domain immunoglobulin suppressor of T cell activation (VISTA or C10ORF54) is a type 1 transmembrane protein that blocks T-cell activation (47). We found that the overall survival and recurrence-free survival was significantly lower in the high-risk group HCC patients with high VISTA expression. Another study showed that VISTA was overexpressed in oral squamous cell carcinoma and correlated with other immune checkpoint markers PD-L1 and CTLA-4. In addition, the study also showed a poor prognosis in patients with high VISTA and low CD8^+^ T-cells (48).

The glycophosphatidylinositol-anchored receptor enzyme, ecto-5′-nucleotidase (CD73 or NT5E) inhibits T-cell receptor activation when adenosine binds to its receptor (49). Our study showed NT5E positivity was a statistically significant predictor of poor overall survival and recurrence-free survival in HCC. Our study is consistent with previous studies in triple negative breast cancer, head and neck squamous cell carcinoma, ovarian cancer, and various other gastric carcinoma where NT5E expression in tumor tissues was correlated with poor prognosis (50–54).

T-cell immunoglobulin and mucin domain-containing-3 (TIM-3 or HAVCR2) is an immune checkpoint receptor that binds to its ligand Galectin-9 and limits the T-cell responses (55). Our study showed that TIM-3 is overexpressed in the high-risk group of HCC patients and had significantly worse overall survival. Another study has also confirmed the high expression of TIM-3 in HCC patient tumors than in healthy controls (56). Furthermore, the overall survival time for patients with higher TIM-3 expression is lower than that of patients with lower TIM-3 expression (57). Taken together, these findings indicate that costimulatory and checkpoint genes can be beneficial for the clinical evaluation of HCC patients, especially to identify patients who are at increased risk of worse survival and relapse. A limitation of our study is the lack of HCC patients treated with immune checkpoint therapies. Further studies to validate the expression of these immune predictors in HCC patient cohorts treated with immune checkpoint therapies will be important. The role of these genes in HCC has not been fully elucidated. However, it is conceivable that these immune regulatory molecules may play pivotal roles in modulating the immune response in HCC. Expression, distribution, and function of these immune regulatory molecules in HCC tissues warrant further investigation.

While the clinical relevance of immune-regulators expressed on immune cells is well established, this study focused on the altered expression of immune regulatory genes in HCC tumors. In addition to serving as useful prognostic biomarkers for HCC, targeting B7-H4, PD-L2, TIM-3, VISTA, CD73, and PD-L1 axis with antagonistic antibodies may prove to be beneficial in a subset of HCC patients with elevated levels of these genes. VTCN1, HAVCR2, NT5E, LGALS9, CD80, and PD-1 axis may also represent useful prognostic biomarkers for HCC. Additionally, elevated VTCN1, HAVCR2, LGALS9, TNFRSF14, and CTLA-4 axis can also be beneficial as prognostic biomarkers for HCC. Given that ICI depend on the receptor–ligand interactions between T-cells and tumor cells, and the combined elevated expression of immune regulatory molecules on tumor-infiltrating T-cells and tumor cells is more predictive of ICI response, further comprehensive studies are needed to address the relationship of these immune regulatory molecules on both tumor and tumor-infiltrating T-cells. A recent study showed improved survival in patients with high chronic inflammatory response in the stroma (58). In support of these findings, clarifying the immune regulators involved in the effector functions of tumor-associated T-cells has important implications for our understanding of how the immune microenvironment is modulated to promote antitumor immune responses.

Although there is interest in the use of ICIs in HCC, the coordinated upregulation of immune checkpoint and other immune-regulated genes in our study suggests that a combinatorial treatment strategy is likely to be more beneficial. Early trial results on the combination of PD-L1 and CTLA-4 targeting were first found to be valuable in malignant melanoma (59). Subsequently, combination of these ICIs also resulted in remarkable tumor regression and improved overall survival in many cancers (60). These clinical trials showed a significant advantage of combination therapy over ICI monotherapies. Recent studies have shown that upregulation of immune-related molecules such as TIM-3 occurs in mice and humans following PD-1 inhibition (61) and in the case of anti-CTLA-4 treatment, VISTA, and PD-L1 were upregulated (62). The elevation in these additional immune regulatory molecules has been proposed to lead to development of resistance to ICI therapies resulting in a significant fraction of cancer patients who do not benefit from the existing checkpoint inhibitor therapies. These findings provide a clinical incentive to combine different ICI therapies to potentially sensitize HCC tumors. In our study, the coordinated expression of immune regulatory molecules, such as *B7-H4, TIM-3*, and *VISTA* with *PD-L1* correlated with poor prognosis, while the co-occurrence of *B7-H4, TIM3, VISTA, CD73*, and *PD-L2* with *PD-L1* correlated with poor recurrence-free survival. The identification of these additional immune biomarkers can help to select patients who might benefit from combination immunotherapy.

Our study is the first to provide direct evidence that EMT phenotype is associated with PD-L1 expression in HCC patient tissues. This observation is in line with another study in pulmonary adenocarcinoma where an association between the messenger RNA EMT signature and high PD-L1 expression was found (24). Another study demonstrated a molecular link between EMT and PD-L1 regulation, in both *in vitro* and *in vivo* models (63). It has been suggested that EMT and PD-L1 may bidirectionally influence each other to promote tumor aggressiveness (64). It is conceivable that HCC patients with EMT phenotype would likely benefit from PD-1/PD-L1 targeted immunotherapy. Further studies of the precise molecular mechanisms underlying the association between EMT and PD-L1 expression in HCC tumor microenvironment are warranted.

Recently, high TMB has been associated with better outcome parameters, such as higher response rates to immunotherapy, longer progression-free survival, and overall survival in melanoma and non-small cell lung cancer (65, 66). A study reported that TMB was more reliable in predicting response rate than the expression of PD-L1 by immunohistochemistry (67). A recent study demonstrated that TMB was a reliable biomarker for predicting response to single checkpoint inhibitor, whereas, outcome after anti-PD-1/PD-L1/anti-CTLA4 combinations appeared to be independent of TMB. Our data suggest that TMB can be used to stratify HCC patients for ICI therapy (66). A limitation of our study is the lack of patients treated with ICIs. Further studies are needed to confirm the relationship between TMB and outcome in immunotherapy-treated HCC patients. Moreover, further understanding of the molecular mechanisms which lead to high TMB in HCC is important. In addition to immune markers and TMB, data are emerging on future development of new predictive biomarker strategies for ICI-based immunotherapy, including tumor-infiltrating lymphocytes, immune gene signatures, and multiplex immunohistochemistry (37).

The immune biomarker research represents a promising strategy to guide patient selection and predicts response to immune checkpoint blockade therapies in terms of durable responses or survival benefit. Blockade of immune regulatory molecules identified in this study, including B7-H4, VISTA, CD73, PD-L2, and TIM-3 can potentially offer a treatment strategy to reinstate host immune response against HCC and ultimately tumor regression. Furthermore, the potential to reverse resistance to ICI depends on proper combination therapy that targets the antitumor immune response. Although a combinatorial approach is likely to be more beneficial, their use may be limited by a risk of developing more side effects with combination therapy. The translation of combination therapy approaches for better clinical success in HCC patients can be improved through further mechanistic insights on immunotherapy combination strategies along with immune biomarkers.

## Author Contributions

RS, PP, and AJ performed data acquisition, analysis, and interpretation. PP performed statistical analysis. RS, AJ, PP, and BD wrote the manuscript. KB, DC, and JS critically revised the manuscript. JS and AJ designed the study. MA performed TMB analysis. RS, AJ, PP, KB, and MA revised the manuscript.

## Conflict of Interest Statement

The authors declare that the research was conducted in the absence of any commercial or financial relationships that could be construed as a potential conflict of interest.

## Footnotes

^1^http://cbioportal.org.

^2^http://bioinformatica.mty.itesm.mx/SurvExpress.

^3^http://www.genecards.org/.

## Abbreviations

HCC, hepatocellular carcinoma; ICI, immune checkpoint inhibitor; PD-1, programmed cell death protein 1; PD-L1, programmed death receptor ligand-1; CTLA-4, cytotoxic T lymphocyte-associated antigen-4, EMT, epithelial-to-mesenchymal transition; PI, prognostic index; VTCN1, V-set domain-containing T-cell activation inhibitor 1; BTLA, B and T lymphocyte attenuator; VISTA, V-domain immunoglobulin suppressor of T cell activation; NT5E, ecto-5′-nucleotidase; TIM-3, T-cell immunoglobulin and mucin domain-containing-3.
